# Looking beyond the neonatal: what does administrative data tell us about the preschool health and developmental outcomes of children exposed to opioids in utero?

**DOI:** 10.23889/ijpds.v9i5.2581

**Published:** 2024-09-18

**Authors:** Rhiannon Owen, James Rafferty, Jane Lyons, Ronan Lyons, Keith Abrams

**Affiliations:** 1 Swansea University; 2 University of Warwick

## Background and Objectives

Healthcare decision-making has previously focused on developing recommendations for single conditions. This research aimed to develop a modeling framework to estimate the effects of multi-indication treatment in people living with multiple long-term conditions.

## Approach

Using a case study in type 2 diabetes mellitus [T2DM], chronic kidney disease [CKD], and heart failure [HF], age-adjusted multistate models were used to estimate the effect of sodium-glucose cotransporter-2 (SGLT2) inhibitors as a multi-indication treatment. Baseline hazards were estimated using population-scale, individual-level, linked anonymized electronic health record (EHR) data for 613,195 individuals aged 55 to 85 years in Wales over a 20-year period. Hazard ratios for treatment effects compared to standard of care were obtained from randomized controlled trials for combinations of multiple-long term conditions and incorporated in a patient-level simulation.

## Results

SGLT2 inhibitors increased the estimated mean life expectancy from 0.02 (95% Confidence Interval (CI): -0.02, 0.05) to 1.44 (95% CI: 1.12, 1.74) years in people living with coexisting T2DM, CKD, and HF. The estimated gain in life expectancy was less than 0.5 years for individuals with T2DM, CKD, and HF in different temporally ordered sequences. The estimated mean time to develop HF increased by 3.1 (95% CI: 3.00, 3.24) and 1.34 (95% CI: 1.12, 1.56) years in individuals with CKD, and in those with CKD followed by T2DM.

## Conclusions and Implications

There is an ever increasing need to appraise interventions in people living with multiple long-term conditions to identify optimal treatment strategies and reduce polypharmacy. Multistate models applied to linked EHRs allow for a more rigorous assessment of treatment effects to inform healthcare policy.

